# Efficacy of virtual reality exercise in knee osteoarthritis rehabilitation: a systematic review and meta-analysis

**DOI:** 10.3389/fphys.2024.1424815

**Published:** 2024-06-19

**Authors:** Wei Wei, Haiting Tang, Yu Luo, Shichang Yan, Qipei Ji, Zhixiang Liu, Huaqiang Li, Fei Wu, Shenqiao Yang, Xin Yang

**Affiliations:** ^1^ School of Health Preservation and Rehabilitation, Chengdu University of Traditional Chinese Medicine, Chengdu, China; ^2^ School of Foreign Languages, Chengdu University of Traditional Chinese Medicine, Chengdu, China

**Keywords:** virtual reality, knee osteoarthritis, systematic reviews, meta-analysis, rehabilitation

## Abstract

**Background:**

This systematic review and meta-analysis aims to investigate the effects of virtual reality (VR) exercise compared to traditional rehabilitation on pain, function, and muscle strength in patients with knee osteoarthritis (KOA). Additionally, the study explores the mechanisms by which VR exercise contributes to the rehabilitation of KOA patients.

**Methods:**

We systematically searched PubMed, the Cochrane Library, Embase, Web of Science, Scopus, and PEDro according to the Preferred Reporting Items for Systematic Reviews and Meta-Analyses (PRISMA) guidelines. Our search spanned from the library construction to 24 May 2024, focusing on randomized controlled trials Primary outcomes included pain, Western Ontario and McMaster Universities Osteoarthritis Index (WOMAC), and muscle strength. Meta-analysis was conducted using RevMan (version 5.4) and Stata (version 14.0). The bias risk of included studies was assessed using the Cochrane RoB 2.0 tool, while the quality of evidence was evaluated using the Grading of Recommendations, Assessment, Development, and Evaluation (GRADE) approach.

**Results:**

This meta-analysis and systematic review included nine studies involving 456 KOA patients. The results indicated that VR exercise significantly improved pain scores (SMD, −1.53; 95% CI: −2.50 to −0.55; *p* = 0.002), WOMAC total score (MD, −14.79; 95% CI: −28.26 to −1.33; *p* = 0.03), WOMAC pain score (MD, −0.93; 95% CI: −1.52 to −0.34; *p* = 0.002), knee extensor strength (SMD, 0.51; 95% CI: 0.14 to 0.87; *p* = 0.006), and knee flexor strength (SMD, 0.65; 95% CI: 0.28 to 1.01; *p* = 0.0005), but not significantly for WOMAC stiffness (MD, −0.01; 95% CI: −1.21 to 1.19; *p* = 0.99) and physical function (MD, −0.35; 95% CI: −0.79 to −0.09; *p* = 0.12).

**Conclusion:**

VR exercise significantly alleviates pain, enhances muscle strength and WOMAC total score in KOA patients, but improvements in joint stiffness and physical function are not significant. However, the current number of studies is limited, necessitating further research to expand on the present findings.

**Systematic review registration:**

https://www.crd.york.ac.uk/prospero/display_record.php?ID=CRD42024540061, identifier CRD42024540061

## 1 Introduction

Osteoarthritis is the most common joint disease, characterized by changes in cartilage, bone hypertrophy, and the formation of bone spurs, affecting over 7% of the global population (Mandl, 2019; Hunter et al., 2020). The knee joint is the most frequently affected, with more than 260 million people suffering from KOA, resulting in significant health and societal costs. As a degenerative musculoskeletal disease, KOA’s incidence rises with advancing age, exacerbating related societal healthcare challenges and necessitating comprehensive therapeutic interventions. Beyond the age of 45, KOA incidence escalates substantially every decade, a trend accentuated by the rapid global aging population (Allen et al., 2022). Consequently, effective methods are urgently needed to promote nuanced treatment and rehabilitation for KOA patients. The fundamental objectives within clinical treatment paradigms entail alleviating pain, slowing disease progression, and enhancing knee joint functionality (Hunter and Bierma-Zeinstra, 2019). For severe KOA cases, intervention measures typically encompass single-joint or total knee arthroplasty (Katz et al., 2021; Hannon et al., 2023), despite the considerable economic burden and postoperative challenges associated with surgical modalities. This issue is particularly pronounced in the elderly population, as postoperative persistent pain and inadequate recovery are common dilemmas (Old et al., 2017), further exacerbated by the potential pain-weakness-pain vicious cycle. In light of these challenges, non-surgical therapies hold a paramount position in contemporary treatment guidelines for non-critical KOA cases (Palmer et al., 2019; Duong et al., 2023). In this context, exercise therapy emerges as a primary and highly effective treatment modality (Kolasinski et al., 2020; Petrigna et al., 2022). However, traditional exercise therapies necessitate on-site treatment at healthcare facilities, posing requirements in terms of time, motivation, and financial resources. Additionally, home-based exercise regimens, while economically practical, often fall short in compliance and efficacy due to challenges in supervision and resource accessibility (Karasavvidis et al., 2020; Cinthuja et al., 2022; Bertolazzi et al., 2024). To address the challenges of enhancing multidimensional patient training, VR technology emerges as a promising solution (Chen et al., 2021).

VR is an innovative technology defined as “interactive simulations created using computer hardware and software to provide users with immersive experiences, allowing them to engage in environments closely related to real-world objects and events” (Lin et al., 2024). The two fundamental elements of VR are immersion and presence. VR devices and systems can be classified into two main types: (1) semi-immersive or non-immersive VR systems; and (2) fully immersive VR systems. Non-immersive VR is achieved through 2D display screens, enabling users to interact with the virtual environment from an “external” perspective while maintaining awareness of the actual environment (Fusco and Tieri, 2022). Semi-immersive VR typically integrates a large display screen for projecting the virtual environment. Users interact with the virtual environment through advanced interface devices while perceiving the real-world environment, resulting in partial immersion and strong presence (Salatino et al., 2023). Fully immersive VR is typically achieved through head-mounted displays (HMDs), which isolate users from the external environment, immersing them in a three-dimensional environment. This sense of “being there” is intensified during immersive experiences, allowing users to interact with the virtual environment using their bodies (Helou et al., 2023). Research suggests that during VR gaming, systolic and diastolic blood pressure may slightly increase, thus implying that engaging in positive VR gaming experiences is akin to a moderate-intensity exercise regimen (Sauchelli and Brunstrom, 2022). Additionally, VR can create a rehabilitation environment where users undergo specified exercises while receiving assessments, thereby stimulating their motivation for extensive practice—all of which are integral components of the rehabilitation process (Saeedi et al., 2021). The efficacy of VR-based exercise therapies has been demonstrated in diseases such as multiple sclerosis, burns, Parkinson’s disease, stroke, and cerebral palsy (Castellano-Aguilera et al., 2022; Liu et al., 2022; Lan et al., 2023; Shen et al., 2023; Yu et al., 2023). In the field of orthopedic rehabilitation, investigations into the advantages of VR-based exercise have been conducted for neck pain, ankle injuries, low back pain, and chronic musculoskeletal disorders (Brea-Gómez et al., 2021; Elaraby et al., 2023; Guo et al., 2023; Kantha et al., 2023). However, there is currently insufficient evidence to prove the benefits of VR in KOA rehabilitation. A review (Byra and Czernicki, 2020) noted that the evidence regarding the superiority of VR-based interventions over standard physical therapy in the rehabilitation of osteoarthritis patients (including those undergoing total knee arthroplasty) is inconclusive. Therefore, this systematic review and meta-analysis evaluate and analyze the rehabilitative effects of VR-based exercise for KOA patients.

## 2 Methods

This systematic review and meta-analysis is prospectively registered with PROSPERO under registration number CRD42023471180. We ensure strict adherence to the standards set forth in the Preferred Reporting Items for Systematic Reviews and Meta-Analyses Statement (PRISMA 2020) (Page et al., 2021) (Supplementary Table S1).

### 2.1 Search strategy

From the establishment of the database to 24 May 2024, two reviewers (W.W. and S.C.Y.) independently conducted comprehensive searches of various databases, including PubMed, the Cochrane Library, Embase, Web of Science, Scopus, and PEDro, to identify potentially relevant studies. The keywords and subject phrases included “knee osteoarthritis” or “KOA” and “virtual reality” or “Virtual Reality Exposure Therapy” or “VR.” Additionally, we manually searched the references of relevant articles and further explored relevant articles via Google Scholar/Google. The complete search strategies for all databases are detailed in Supplementary Table S2.

### 2.2 Study selection

Two reviewers (Z.L. and Q.J.) independently screened articles for eligibility based on the following criteria. Eligibility criteria based on the PICOS guidelines (Higgins et al., 2019) (participants, interventions, comparisons, outcomes, and study design) were as follows: (1) Participants: patients with a confirmed diagnosis of KOA, irrespective of age and gender; (2) Interventions: VR exercise therapy used or VR exercise therapy combined with conventional rehabilitation; (3) Comparison groups: received conventional rehabilitation; (4) Outcomes: pain scores, WOMAC and muscle strength; (5) Study design: only RCTs were included. Exclusion criteria included: (1) use of VR only as an assessment tool; (2) retrospective studies, reviews, case reports, and conference abstracts; (3) incomplete article data, full text unavailable after contacting the corresponding author; (4) duplicate publications; and (5) video games using non-VR media. Articles published in languages other than English were translated and included if they met the eligibility criteria. Disagreements that arose during the screening process were discussed repeatedly to reach consensus. Persistent disagreements are referred to a third party (S.Q.Y.) for resolution.

### 2.3 Data extraction

Two researchers (H.L. and Y.L.) autonomously conducted literature screening based on predetermined inclusion and exclusion criteria. Data extraction was performed independently using a pre-designed standardised data extraction sheet in Microsoft Excel. Extracted data included basic details of the included trials, such as lead author, country, and year of publication; basic characteristics of the study participants, including the number of patients, age distribution, and gender composition; specifics of the intervention, such as VR equipment used, level of immersion stem, and duration of the intervention; and results of the main rehabilitation assessments, including pain scores, WOMAC and muscle strength. Corresponding author (X.Y.) requested missing data by email. If no answer was received, the study was excluded from the review. Inter-assessor discrepancies were resolved through discussion or input from a third assessor (S.Q.Y.).

### 2.4 Risk of bias assessment

We assessed the bias risk of each eligible study using the Cochrane RoB 2.0 tool (Sterne et al., 2019), which evaluates risk of bias in five key areas: randomization process, deviations from intended interventions, missing outcome data, measurement of the outcome, and selection of the reported result. Scoring was done independently by two reviewers (W.W. and H.T.). In case of inconsistent results, they were discussed with a third reviewer (S.Q.Y.). Any disagreements were resolved through consultation with a third party (S.Q.Y.).

### 2.5 Certainty of the evidence

The certainty of evidence was appraised employing the Grading of Recommendations, Assessment, Development, and Evaluation (GRADE) methodology, facilitated by the online GRADEpro app (GRADEpro). Each outcome underwent scrutiny concerning limitations, inconsistency, indirectness, imprecision, and publication bias (Guyatt et al., 2011). The certainty of evidence was stratified into categories of “high,” “moderate,” “low,” or “very low” (Balshem et al., 2011).

### 2.6 Statistical methods

Meta-analyses were performed using RevMan 5.4 and forest plots were generated. All extracted data were entered and checked by reviewers (W.W. and H.T.). In our study, all included outcomes were continuous variables, so we used the mean difference (MD) with a 95% confidence interval (CI) to calculate the overall effect of VR-based exercise. Standardised MDs (SMDs) were used if studies used different measures, units or grading systems to express results. Heterogeneity was assessed by the chi-square test (test level α = 0.10) and quantitatively using the I^2^ test (Cumpston et al., 2019). If I^2^ < 50% and *p* > 0.1, data were combined using a fixed-effects model. Where I^2^ > 50% and *p* ≤ 0.1 indicated a high degree of heterogeneity, meta-analyses were performed using a random effects model. In addition, this study referenced the criteria specified in recent literature (Concoff et al., 2019; Kim et al., 2021), comparing the minimal clinically important differences (MCID) for pain and WOMAC scores. Subgroup analyses were used to compare the efficacy of VR on pain, and WOMAC scores. Sensitivity analyses were performed by removing each study individually via Stata 14.0, and studies were considered influential if removal of a study significantly altered the combined effect.

### 2.7 Publication bias

Egger’s test and the generation of funnel plots were not used to evaluate publication bias because there were fewer than 10 articles for each of the combined outcomes in this systematic review and meta-analysis (Cumpston et al., 2022).

## 3 Results

### 3.1 Search results

**FIGURE 1:**
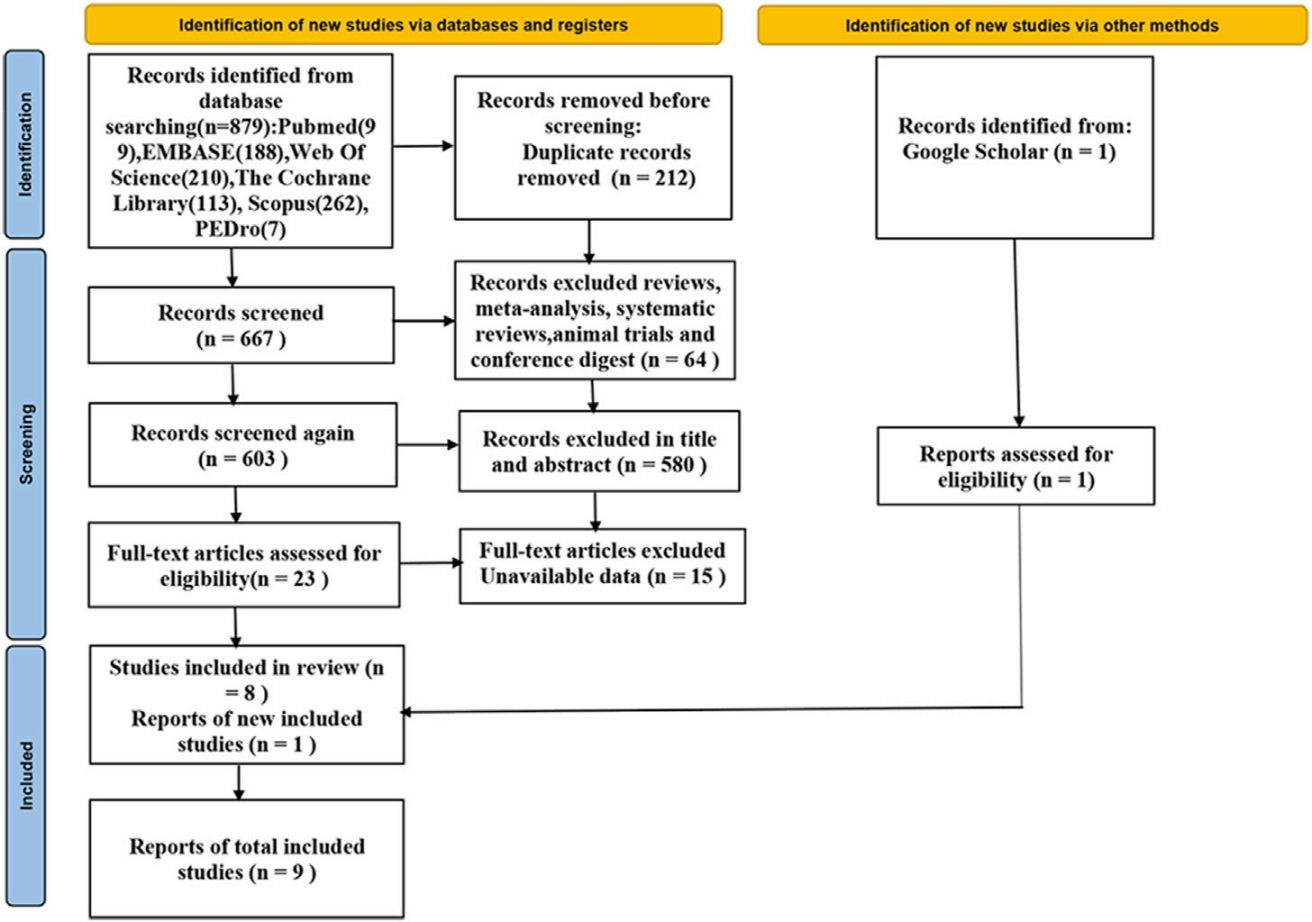
Flow chart of study selection according to PRISMA guidelines.

In the initial database search, 879 relevant articles were found. After removal of duplicate entries, the remaining corpus consisted of 667 articles. Articles categorised as reviews, systematic reviews, meta-analyses and animal experiments were subsequently excluded, as well as articles where a lack of consistency in the research was identified after careful examination of titles and abstracts. A total of 23 articles were screened through this meticulous collation process. As complete data were still not available after contacting the corresponding authors, we further refined the articles, and after including one article from Google Scholar, we ultimately retained nine articles that met specific criteria (Lin et al., 2007; Abdelazeem et al., 2016; Kim et al., 2017; Lin et al., 2020; Nambi et al., 2020; Lin et al., 2022; Mete and Sari, 2022; Ozlu et al., 2023; Oliveira et al., 2024). The detailed literature screening process is shown in Figure 1.

### 3.2 Study characteristics

Table 1 provides a comprehensive summary of the nine RCTs included in this systematic review and meta-analysis. These trials recruited a total of 456 patients with KOA across different age groups. The age distribution ranged from 20 to 70 years, with 229 participants receiving VR–based rehabilitation therapy and 227 participants receiving traditional rehabilitation therapy. Regarding the severity of KOA, four studies (Abdelazeem et al., 2016; Nambi et al., 2020; Mete and Sari, 2022; Ozlu et al., 2023) included patients with Kellgren-Lawrence (K-L) grade 2 to 3, one (Oliveira et al., 2024) study included patients with K-L grade 2 to 4, one study (Lin et al., 2022) included patients with K-L grade 2, one study (Kim et al., 2017) did not specify the severity of KOA in the recruited patients, and two studies included patients with K-L grade ≥2 (Lin et al., 2020) and ≤3 (Lin et al., 2007), respectively. These studies were conducted across various countries and regions, including two in Turkey (Mete and Sari, 2022; Ozlu et al., 2023), two in Saudi Arabia (Abdelazeem et al., 2016; Nambi et al., 2020), one in Brazil (Oliveira et al., 2024), one in South Korea (Kim et al., 2017), and three in China (Lin et al., 2007; Lin et al., 2020; Lin et al., 2022). The immersion level of VR therapy included non-immersive and immersive modalities, with five studies (Lin et al., 2007; Kim et al., 2017; Lin et al., 2020; Lin et al., 2022; Oliveira et al., 2024) utilizing non-immersive VR and four studies (Abdelazeem et al., 2016; Nambi et al., 2020; Mete and Sari, 2022; Ozlu et al., 2023) utilizing immersive VR. In terms of treatment duration, two studies (Lin et al., 2020; Nambi et al., 2020) had a treatment duration of 4 weeks, four studies (Lin et al., 2007; Abdelazeem et al., 2016; Kim et al., 2017; Oliveira et al., 2024) had a duration of 8 weeks, one study (Lin et al., 2022) had a duration of 12 weeks, and two studies had durations of 3 weeks (Ozlu et al., 2023) and 6 weeks (Mete and Sari, 2022), respectively. Regarding treatment frequency, three studies (Nambi et al., 2020; Lin et al., 2022; Oliveira et al., 2024) had a frequency of two sessions per week, four studies (Lin et al., 2007; Abdelazeem et al., 2016; Kim et al., 2017; Lin et al., 2020) had a frequency of three sessions per week, and two studies (Mete and Sari, 2022; Ozlu et al., 2023) had a frequency of five sessions per week.

**TABLE 1:** Characteristics of included studies.

Study (year)	Country	Sample size (n)	Sex (M/F)	Age mean (SD) year	K-L grade	VR equipment	Degree of immersion	Duration of rehabilitation	Number of treatments	Treatment time(min)	Outcome
Lin et al. (2020)	China	EG: 40	EG: 16/24	EG: 55.9 (15.8)	≥2	Hot Plus system	Non-immersive	4 weeks	3/week	20	WOMAC, Chronic Pain Scale
		CG: 40	CG: 23/17	CG: 58.1 (16.9)							
Nambi et al. (2020)	Saudi Arabia	EG: 20	NA	EG: 22.8 (1.3)	2–3	Pro-Kin system	Immersive	4 weeks	2/week	20	VAS, WOMAC
		CG: 20		CG: 22.6 (1.4)							
Mete and Sari (2022)	Turkey	EG: 30	EG: 6/24	EG: 59.5 (7.0)	2–3	MarVAJED^®^	Immersive	6 weeks	5/week	20	VAS, WOMAC
		CG: 30	CG: 7/23	CG: 57.7 (10.9)							
Ozlu et al. (2023)	Turkey	EG: 35	EG: 18/17	EG: 53.28 (10.42)	2–3	the Quest 128 GB type of Oculus brand	Immersive	3 weeks	5/week	15	VAS, WOMAC
		CG: 38	CG: 12/26	CG: 53.71 (9.65)							
Kim et al. (2017)	Korea	EG: 15	EG: 7/8	EG: 76.5 (8.8)	NA	Shinhwa, MX-0004SE	Non-immersive	8 weeks	3/week	15	Muscle strength
		CG: 15	CG: 5/10	CG: 77.7 (7.9)							
Lin et al. (2022)	China	EG: 20	EG: 1/19	EG: 75.6 (4.4)	2	CRE system and LabVIEW software	Non-immersive	12 weeks	2/week	30	WOAMC, Muscle strength
		CG: 18	CG: 2/16	CG: 76.0 (5.6)							
Lin et al. (2007)	China	EG: 29	EG: 9/20	EG: 61.6 (8.1)	≤3	Acer system	Non-immersive	8 weeks	3/week	40	WOMAC, Muscle strength
		CG: 26	CG: 5/21	CG: 61.0 (7.7)							
Abdelazeem et al. (2016)	Saudi Arabia	EG: 20	NA	EG: 58 (6.0)	2–3	Xbox 360 with Kinetic Sensor	Immersive	8 weeks	3/week	15	VAS, WOMAC
		CG: 20		CG: 59 (7.0)							
Oliveira et al. (2024)	Brazilian	EG: 20	EG: 6/14	EG: 62.35 (7.39)	2–4	Xbox 360 with Kinetic Sensor	Non-immersive	8 weeks	2/week	20	VAS, WOMAC
		CG: 20	CG: 3/17	CG: 62.60 (8.62)							

EG, experimental group; CG, control group; NA, not available; K-L grade, Kellgren-Lawrence Grade; SD, standard deviation; VAS, visual analogue scale; WOMAC, Western Ontario and McMaster Universities Osteoarthritis Index; 10MWT., 10-m walk test; MMT, manual muscle test.

### 3.3 Risk of bias

**FIGURE 2:**
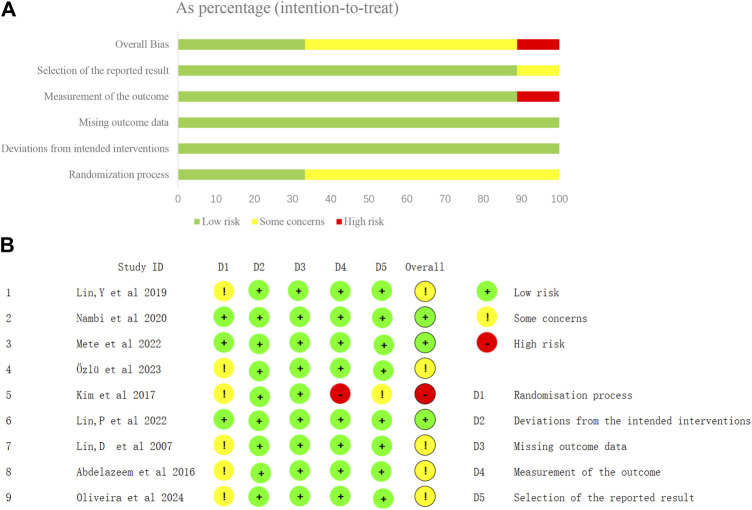
Risk-of-bias graph and summary. **(A)** Overall risk of bias, with each category presented as percentages. **(B)** Risk of bias of the studies included in the systematic review.

In total, nine studies were considered to have problems with overall bias, with one study (Kim et al., 2017) having a high risk of bias and three studies (Nambi et al., 2020; Lin et al., 2022; Mete and Sari, 2022) having a low risk of bias (Figures 2A, B). Some problems in the first area (deviation from the intended intervention) were caused by the fact that several of the included studies did not mention whether the allocation method was hidden or not. The nature and setting of the intervention made it difficult to blind the patients or therapists to the intervention, with most trials only being able to blind the outcome measures, and only two studies (Nambi et al., 2020; Mete and Sari, 2022) blinding the patients and therapy separately. The risk of bias in a study’s outcome measures was high because of insufficient information for blinded assessments (Kim et al., 2017).

### 3.4 Results of the meta-analysis

#### 3.4.1 Pain

**FIGURE 3:**
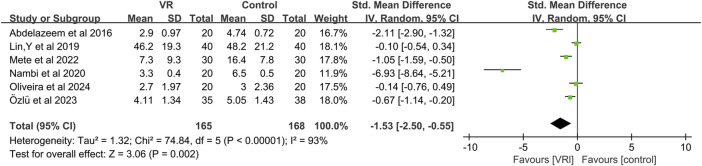
Forest plot for VR-based exercise compared with controls in pain.

**FIGURE 4:**
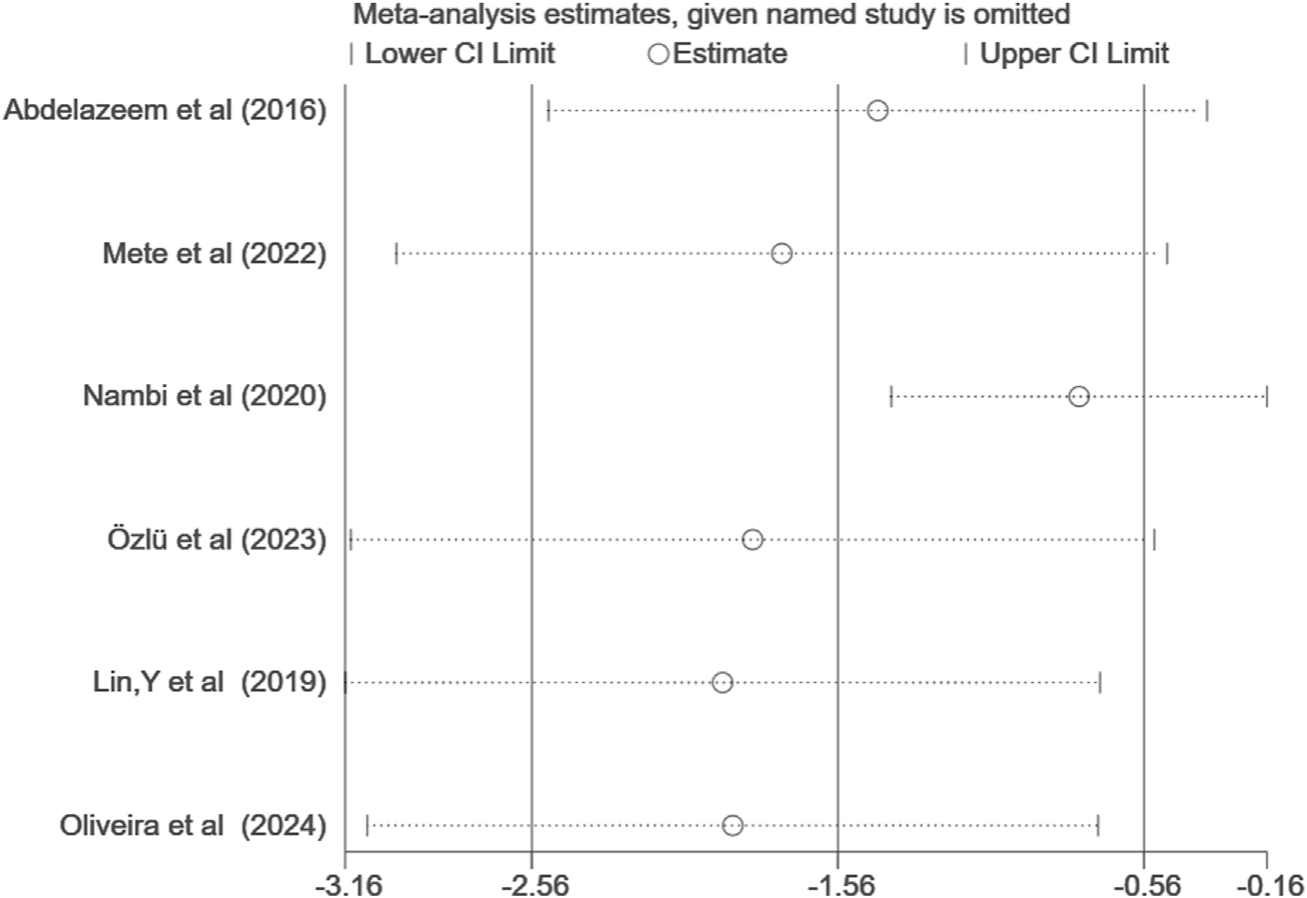
Sensitivity analysis of pain.

Six studies (Abdelazeem et al., 2016; Lin et al., 2020; Nambi et al., 2020; Mete and Sari, 2022; Ozlu et al., 2023; Oliveira et al., 2024) evaluated changes in pain scores, with Lin,Y et al. (Lin et al., 2020) employing the Chronic Pain Scale (0–100 points), while the remaining five studies (Abdelazeem et al., 2016; Nambi et al., 2020; Mete and Sari, 2022; Ozlu et al., 2023; Oliveira et al., 2024) utilized the Visual Analog Scale (VAS) (0–10 points). Given the different measurement methods, we utilized the SMD to calculate the overall effect. Due to significant heterogeneity (I^2^ = 93%), we employed a random-effects model to conduct a meta-analysis of the six articles (involving 333 participants). The results indicated that VR-based exercise therapy significantly improved pain in patients with KOA compared to traditional rehabilitation treatment, and the difference was statistically significant (SMD, −1.53; 95% CI: −2.50 to −0.55, *p* = 0.002) (Figure 3). Upon comparison, VR exercise reached the previously established MCID level of 1.23 for pain improvement, as determined by prior studies (Concoff et al., 2019). Following sensitivity analysis (Figure 4), exclusion of three studies (Abdelazeem et al., 2016; Nambi et al., 2020; Mete and Sari, 2022), resulted in a significant reduction in overall effect size heterogeneity (I^2^ = 42%). However, the overall effect size remained non-significantly changed (SMD, −0.31; 95% CI: −0.69 to 0.07, *p* = 0.11).

##### 3.4.1.1 Subgroup analysis

As shown in Table 2 (Supplementary Figure S1), we conducted subgroup analyses based on the immersion level of VR, treatment duration, treatment frequency, and intensity of VR exercise. Grouping by the immersion level of VR, we found that immersive VR is more effective in alleviating pain compared to non-immersive VR. Regarding treatment duration, the efficacy of treatments lasting <6 weeks is superior to those lasting ≥6 weeks. Additionally, the best outcomes are observed with treatment frequencies of ≤3 times per week and session intensities of 20 min each.

**TABLE 2:** Subgroup analyses of pain and WOMAC total score.

Outcome or subgroup	Number of studies	SMD (95%CI)	*p*-values	Possible intervention approaches
**Pain**				
Overall	6	−1.53 [ −2.50, −0.55]	**0.002**	Immersive VR, 20 min/session, ≤3 sessions/week, Treatment durations <6 weeks
**Degree of immersion**				
Immersive	4	−2.41 [ −3.89, −0.93]	**0.001**	
Non-immersive	2	−0.11 [ −0.47, 0.25]	0.55	
**Treatment durations**				
<6 weeks	3	−2.25 [ −4.20, −0.29]	**0.02**	
≥6 weeks	3	−1.07 [ −2.09, −0.05]	**0.04**	
**Treatment frequency**				
>3/week	2	−0.83 [ −1.20, −0.47]	**< 0.00001**	
≤3/week	4	−2.10 [ −3.88, −0.32]	**0.02**	
**Treatment time**				
15 min	2	−1.35 [–2.77, 0.06]	0.06	
20 min	4	−1.73 [–3.20, −0.26]	**0.02**	
**WOMAC total score**				
Overall	5	−14.79 [–28.26, −1.33]	**0.03**	Immersive VR, 15 min/session, ≤3 sessions/week, Treatment durations <6 weeks
**Degree of immersion**				
Immersive	4	−17.58 [–32.61, −2.55]	**0.02**	
Non-immersive	1	−2.81 [–11.43, 5.81]	0.52	
**Treatment durations**				
<6 weeks	2	−19.19 [–36.94, −1.44]	**0.03**	
≥6 weeks	3	−11.75 [–33.66, 10.17]	0.29	
**Treatment frequency**				
>3/week	2	−5.82 [–13.78, 2.14]	0.15	
≤3/week	3	−22.40 [–29.94, −14.86]	**< 0.00001**	
**Treatment time**				
15 min	2	−20.14 [–39.78,–0.50]	**0.04**	
20 min	3	−11.12 [–31.70, 9.46]	0.29	

SMD, standardised mean difference, 95%CI: 95% confidence interval, min: minutes. Bold value means p< 0.05.

#### 3.4.2 WOMAC

**FIGURE 5:**
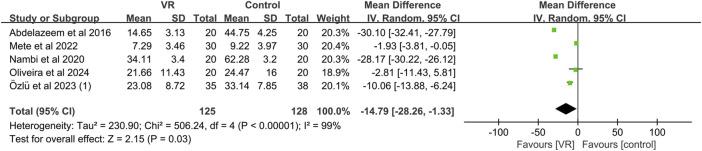
Forest plot for VR-based exercise compared with controls in WOMAC.

**FIGURE 6:**
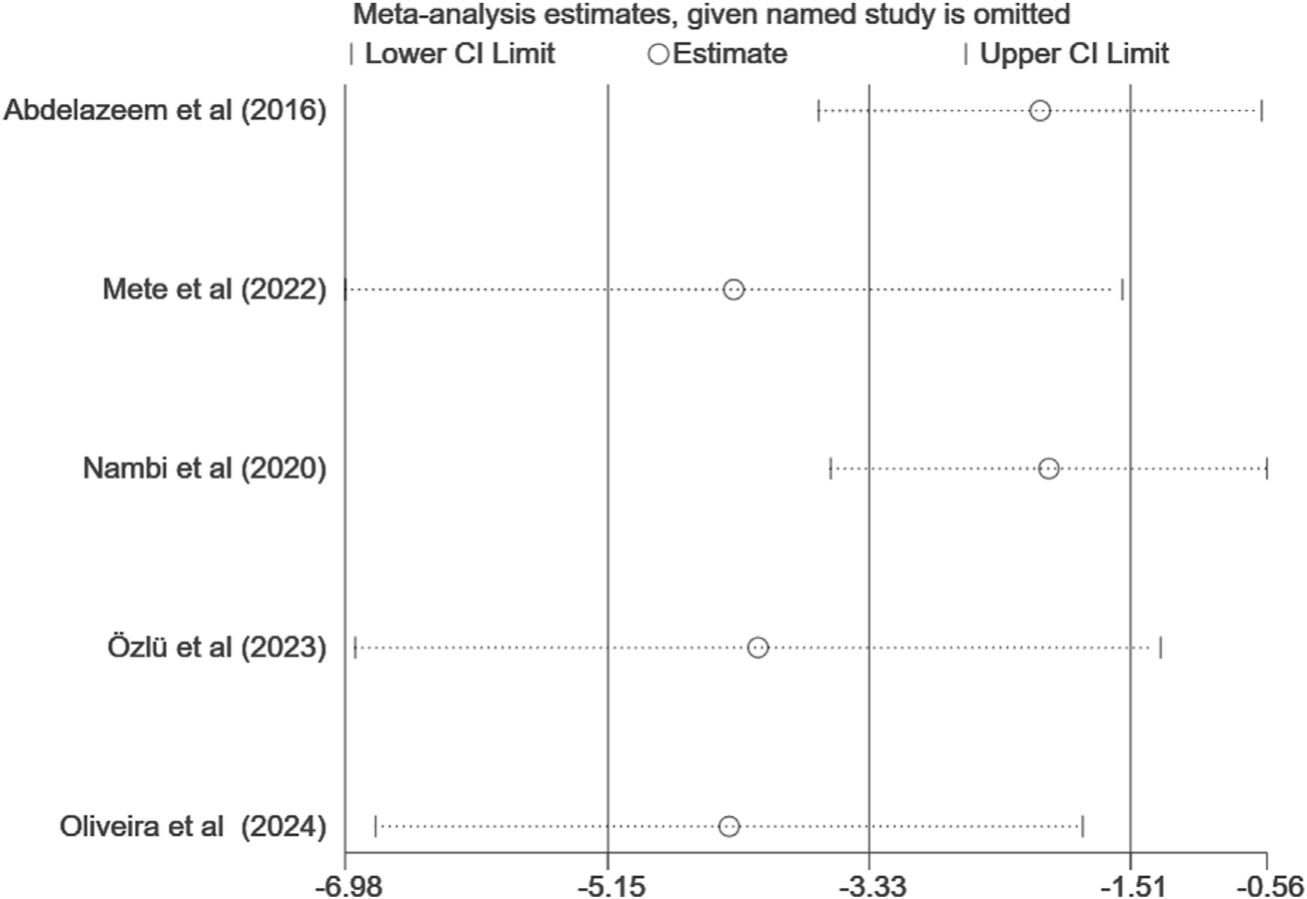
Sensitivity analysis of WOMAC.

Five studies (Abdelazeem et al., 2016; Nambi et al., 2020; Mete and Sari, 2022; Ozlu et al., 2023; Oliveira et al., 2024) reported WOMAC outcomes after VR treatment. Among them, two studies (Lin et al., 2020; Mete and Sari, 2022) reported WOMAC pain, stiffness, and physical function scores separately, but Lin,Y et al. (Lin et al., 2020) did not report the total WOMAC score. Therefore, we did not include this study in the pooled data analysis of the WOMAC total score. Due to significant heterogeneity (I^2^ = 99%), we employed a random-effects model for meta-analysis. As shown in Figure 5, the meta-analysis of WOMAC total scores for 253 KOA patients receiving VR-based exercise therapy demonstrated significant improvement compared to conventional rehabilitation, with statistically significant differences (MD, −14.79; 95% CI: −28.26 to −1.33; *p* = 0.03). However, compared to previous studies (Kim et al., 2021), the WOMAC total score did not reach the MCID level (16.1). To assess the stability of the combined results, sensitivity analysis revealed (Figure 6) that after excluding three studies (Abdelazeem et al., 2016; Nambi et al., 2020; Ozlu et al., 2023), the overall effect size still reached statistical significance (MD, −1.97; 95% CI: −3.81 to −0.13; I^2^ = 0%, *p* = 0.04).

Analysis of WOMAC pain, stiffness, and physical function scores revealed that VR exercise significantly improved the WOMAC pain score (MD, −0.93; 95% CI: −1.52 to −0.34; I^2^ = 0%; *p* = 0.002) compared to conventional rehabilitation, but the improvements in WOMAC stiffness and physical function scores were not significant (MD, −0.01; 95% CI: −1.21 to 1.19; *p* = 0.99); (MD, −0.35; 95% CI: −0.79 to −0.09; *p* = 0.12) (Supplementary Figure S2). In comparison with previous studies, the results indicate that WOMAC pain, stiffness, and physical function scores did not reach the MCID levels (4.2, 1.9, 10.1) (Kim et al., 2021).

##### 3.4.2.1 Subgroup analysis

We also conducted subgroup analyses based on the immersion level of VR, treatment duration, treatment frequency, and intensity of VR exercise. As shown in Table 2 (Supplementary Figure S3), the subgroup analysis indicated that immersive VR is more effective than non-immersive VR in improving WOMAC scores. Additionally, a treatment frequency of ≤3 times per week yielded better results compared to a frequency of >3 times per week. Furthermore, a treatment duration of <6 weeks proved more effective than a duration of ≥6 weeks. In terms of intensity, sessions lasting 15 min were more effective than those lasting 20 min.

#### 3.4.3 Muscle strength

**FIGURE 7:**
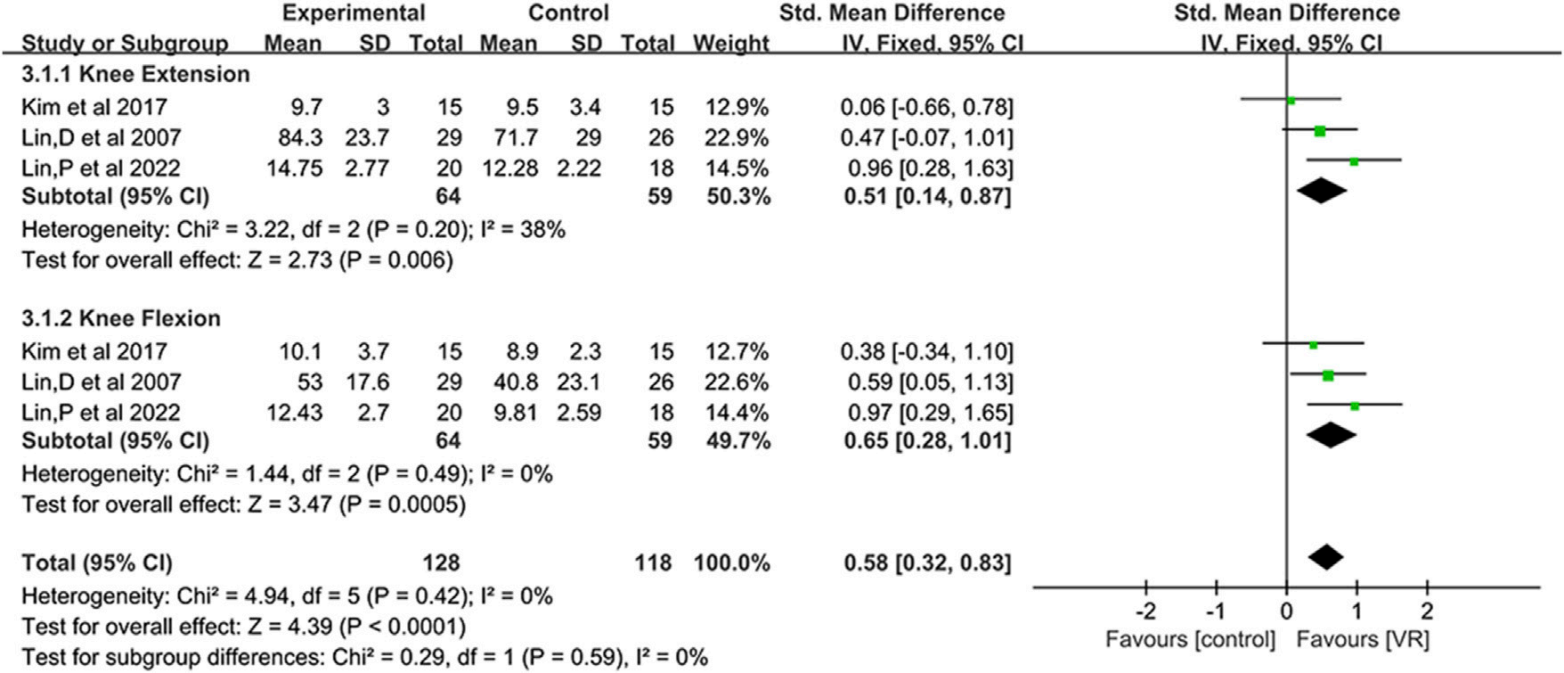
Forest plot for VR-based exercise compared with controls in muscle strength.

Three studies (Lin et al., 2007; Kim et al., 2017; Lin et al., 2022) recorded changes in knee flexor and extensor muscle strength. Due to the use of different units of measurement (kilograms, pounds, and newtons) and unclear heterogeneity (I^2^ = 38%) across these three studies, we employed SMD to calculate the overall effect, conducting a meta-analysis using a fixed-effects model. The results, as shown in Figure 7, compared to traditional rehabilitation therapy, in 64 KOA patients undergoing VR exercise therapy, both knee joint extensor (SMD, 0.51; 95% CI: 0.14 to 0.87; *p* = 0.006) and flexor (SMD, 0.65; 95% CI: 0.28 to 1.01; *p* = 0.0005) strength showed significant improvements. Moreover, the study found that the enhancement of knee joint flexor strength was superior to that of knee joint extensor.

### 3.5 Certainty of the evidence

Due to the inconsistency among included studies, the certainty of evidence for pain score was classified as “moderate,” while the WOMAC total score was deemed “low quality” due to inconsistency and imprecision of the trials. The evidence certainty for knee flexor and extensor muscle strength was defined as “very low” due to the risk of bias, inconsistency, and imprecision of the included studies. Specific GRADE assessments are provided in Supplementary Table S3.

## 4 Discussion

The primary objective of this systematic review and meta-analysis is to assess the existing evidence regarding the efficacy of VR-based exercise for KOA patients. To the best of our knowledge, this is the first systematic review and meta-analysis examining the impact of VR-based exercise therapy on KOA patients. Importantly, VR-based training appears to be an effective modality. Our study results indicate that VR-based exercise therapy can improve pain and WOMAC scores while enhancing knee joint muscle strength, although the improvement in WOMAC stiffness and physical function scores is not significant.

### 4.1 Effects of VR-based exercise on pain

Chronic joint pain is the most common debilitating symptom of OA, affecting not only patients’ physical health but also impacting sleep, mood, and overall quality of life (Kılıçaslan et al., 2023). In KOA patients, alterations in neural network excitability and connectivity shift from regions primarily associated with sensory processing to those associated with emotional processing (Soni et al., 2019). The cortical limbic system plays a crucial role in the initiation, perpetuation, and exacerbation of chronic pain, correlating with the emotional aspects of pain and influencing emotional and motivational responses (Howard et al., 2012). Previous research has explored the role of cognitive-emotional factors in the occurrence and persistence of chronic musculoskeletal pain (Martinez-Calderon et al., 2020; Martinez-Calderon et al., 2022), and KOA pain similarly affects brain areas responsible for sensory discrimination, cognitive processing, and the emotional aspects of pain (Hazra et al., 2022). Recent studies have indicated that during the COVID-19 pandemic, VR has emerged as a widely used therapeutic tool for pain management (Pallavicini et al., 2022). VR modulates pain perception by stimulating visual, auditory, and somatosensory-motor networks (Ahmadpour et al., 2019). Additionally, VR application increases activity in areas associated with pain inhibition. Prior research suggests that VR, by modulating the activity of downstream pain control systems, shifts attention away from pain and influences pain perception (Gold et al., 2007). Our meta-analysis, incorporating recent research findings, concludes that VR-based exercise therapy is effective in improving pain in KOA patients, consistent with prior findings (Byra and Czernicki, 2020). The results of subgroup analysis indicate that immersive VR training has a greater impact on pain compared to non-immersive VR training, consistent with prior research (Malloy and Milling, 2010). This outcome may be attributed to the lower sensory substitution level in non-immersive VR, leading to greater perception of pain. Immersive VR with more immersion may reduce attention to pain perception (Hoffman et al., 2004; Gutierrez-Martinez et al., 2010).

### 4.2 Effects of VR-based exercise on function

KOA-induced joint degradation and inflammation can result in knee joint pain and functional impairment. Abnormal mechanical loading can damage joint cartilage, potentially triggering abnormal cellular activities in cartilage and synovium, ultimately leading to joint stiffness (Lu et al., 2018). WOMAC is one of the commonly used outcome measures to assess function in KOA patients (Collins et al., 2011). Our meta-analysis suggests that VR-based exercise can enhance the functionality of KOA patients. However, in terms of WOMAC stiffness and physical function scores, the improvement effects of VR exercise are not significant; nevertheless, this result is based on only two studies. We acknowledge that the limited number of included studies may compromise the stability and reliability of the results, potentially failing to adequately represent the entire field. The improvement in WOMAC total score can be attributed to pain relief and increased exercise persistence. Despite the beneficial effects of exercise on KOA, chronic pain experiences often lead patients to reduce activity to avoid discomfort, resulting in a significant decline in their joint function (Nazari et al., 2019). Simultaneously, VR exercise increases dopamine release in the striatum (Lin et al., 2020). The enhanced dopamine transmission contributes to improved attention, facilitates sensorimotor integration, and reinforces behavior (Koepp et al., 1998). Through the central integration theory, VR exercise restores knee joint muscle strength by stimulating proprioceptors through sensorimotor integration, thereby improving joint function and stability (Riemann and Lephart, 2002). This could potentially represent one of the physiological mechanisms through which VR exercise improves function. Regular exercise is a crucial component of rehabilitation, yet some patients exhibit poor adherence to exercise persistence (Dobson et al., 2016; Combalia et al., 2024). VR provides a more engaging environment for exercise, with gamified environments highlighting tasks more effectively than blind repetition, significantly enhancing patients’ motivation and compliance in rehabilitation (Alfieri et al., 2022). Moreover, real-time feedback provided by VR training creates a positive environment for patients, enabling rapid activation of muscle kinematics learning (Nambi et al., 2020). Furthermore, VR training activates sensory functions, restoring movement functionality, further improving functional disability conditions.

### 4.3 Effects of VR-based exercise on muscle strength

Meanwhile, the aforementioned improvements in function are accompanied by an enhancement in muscle strength. Robust muscle strength is crucial for KOA patients, as strong muscles can act as shock absorbers to protect joint stability (Bennell et al., 2013). A previous review (Byra and Czernicki, 2020) reported that VR training does not affect muscle strength around the knee joint in KOA patients, while our study suggests that VR exercise can improve knee joint muscle strength. Additionally, in our meta-analysis, improvement in flexor muscle strength was found to be more favorable compared to extensor muscle strength around the knee joint. It is worth noting that hip muscles also play a significant role in supporting stability and controlling movement of the knee joint, and during the progression of KOA, hip muscles may also experience pain and strength decline (Suzuki et al., 2019). Unfortunately, in the studies included in our analysis, there were no reports on the impact of VR training on hip muscle strength.

### 4.4 Effects of VR-based exercise on different K-L grades of KOA

Early KOA patients (K-L grade 2) exhibit minimal joint structural damage, primarily characterized by mild cartilage degeneration and osteophyte formation. Our study (Abdelazeem et al., 2016; Lin et al., 2020; Nambi et al., 2020; Mete and Sari, 2022; Ozlu et al., 2023; Oliveira et al., 2024) shows that patients at this stage respond well to VR-based exercise for pain relief due to the higher plasticity of their nervous systems and lower sensitivity of pain receptors (Ahmadpour et al., 2019). Functionally, early KOA patients generally perform well despite experiencing some pain and stiffness, enabling them to engage in most daily activities. VR-based exercise can significantly improve their range of motion and muscle strength, thereby preventing disease progression. Muscle strength in early KOA patients might only be slightly affected. Given the greater neuromuscular plasticity in early KOA patients, VR therapy effectively enhances muscle strength and improves motor function, thus preventing further joint damage. In patients with mid-stage KOA (K-L grade 3), joint space narrowing, significant cartilage damage, increased functional impairment, and intensified pain are observed. Most of the studies we included (Abdelazeem et al., 2016; Nambi et al., 2020; Mete and Sari, 2022; Ozlu et al., 2023; Oliveira et al., 2024) encompass patients at this grade, demonstrating that VR-based exercise can also effectively alleviate pain in mid-stage KOA patients. Although the effects may not be as pronounced as in early KOA patients, VR therapy still provides significant pain relief by diverting attention and promoting endogenous pain relief mechanisms (Garrett et al., 2017). Additionally, VR can improve function by enhancing the strength and stability of muscles around the knee joint. Although muscle strength recovery in mid-stage KOA patients may not be as marked as in early-stage patients, continuous VR training can significantly improve their motor ability and quality of life. Late-stage KOA patients (K-L grade 4) suffer from severe joint deformity, nearly complete cartilage degeneration, persistent severe pain, and substantial functional impairment. Only one study we included (Oliveira et al., 2024) explicitly involved late-stage KOA patients. The results indicate that VR-based exercise has limited efficacy at this stage due to the complexity of pain sources and the involvement of multiple pathological mechanisms. However, VR therapy can still help patients manage pain and improve their quality of life by providing distraction and psychological support. VR exercise can also partially improve WOMAC scores in late-stage KOA patients, likely due to the psychological support and increased engagement in physical activity, helping them better manage pain and maintain function (Wang et al., 2023). In terms of muscle strength, late-stage KOA patients experience significant muscle strength loss, especially around the hip and knee joints. VR training alone may not fully restore muscle strength. Unfortunately, the studies we included on muscle strength did not involve late-stage patients, highlighting the need for further high-quality research to support this viewpoint. Overall, VR-based exercise positively impacts KOA patients across different K-L grades, although the effects diminish with increasing severity of KOA. The most significant improvements are observed in early and mid-stage KOA patients, while late-stage patients still receive some functional support despite limited effects. These findings underscore the importance of adaptive and personalized treatment strategies at different stages of the disease.

### 4.5 Subgroup analysis and possible interventions of VR-based exercise

The optimal intervention measures utilizing VR exercise for the rehabilitation of KOA patients remain uncertain. Our subgroup analysis indicates that applying immersive VR with a treatment duration of less than 6 weeks, a treatment frequency of ≤3 times per week, and an intensity of 20 min per session has a more significant therapeutic effect on pain in KOA patients. For WOMAC scores, our subgroup analysis suggests that the treatment effect is superior when using immersive VR with a treatment duration of less than 6 weeks, a treatment frequency of ≤3 times per week, and an intensity of 15 min per session. We believe that immersive VR, with a treatment frequency of no more than 3 times per week and a treatment duration of less than 6 weeks, may offer better rehabilitation outcomes for KOA patients. However, we are currently unable to draw conclusions regarding the intensity of VR exercise, and further combined high-quality research is needed to determine the most suitable intensity for VR exercise in KOA patients. These recommendations aim to standardize intervention methods, enhance comparability in future research, and thus provide more robust evidence for the efficacy of VR exercise therapy in patients with KOA. Additionally, it is worth noting that when implementing precise and individualized VR exercise in clinical practice, patient safety factors such as dizziness and falls during training should also be considered.

### 4.6 Limitations of this systematic review

Firstly, the inevitable heterogeneity among included studies, such as variations in geographical regions, age and gender proportions of participants, intervention duration and frequency, and levels of immersion, may potentially impact the scientific validity of the meta-analysis. Secondly, due to the nature of the interventions, it is challenging for included studies to implement double-blinding in methodology. Thirdly, most of the included studies had small sample sizes, which could have some influence on the outcomes. Lastly, considering that KOA is more common in elderly women (Tang et al., 2016), there may be differences in the efficacy of pain management and functional recovery among patients of different genders and age groups, highlighting the need for higher quality studies involving more male participants or different age cohorts.

### 4.7 Implications for clinical practice and future research

The findings of our meta-analysis hold significant implications for clinical practice and the rehabilitation of KOA patients. VR-based exercise therapy emerges as a promising modality for managing pain and improving function in this patient population. Practically, healthcare providers can integrate VR technology into existing rehabilitation programs for KOA patients. By incorporating immersive VR exercises tailored to individual patient needs, clinicians can enhance treatment outcomes by providing engaging and interactive rehabilitation experiences. VR-based interventions offer a novel approach to pain management by diverting attention away from pain perception and promoting adherence to exercise programs. Furthermore, the accessibility and adaptability of VR technology make it suitable for use in various clinical settings, including outpatient clinics, rehabilitation centers, and even home-based therapy programs. Overall, our study underscores the potential of VR-based exercise therapy as a valuable addition to the armamentarium of treatments available for KOA patients. Looking ahead, several avenues for future research in this field warrant exploration. First, longitudinal studies are needed to assess the long-term effects of VR-based exercise therapy on pain, function, and quality of life in KOA patients. Additionally, further investigation is warranted to elucidate the optimal parameters for VR interventions, including immersion level, treatment duration, frequency, and intensity. Comparative studies evaluating the effectiveness of VR therapy in combination with other modalities, such as traditional physical therapy or pharmacological interventions, could provide valuable insights into the synergistic effects of integrated treatment approaches. Furthermore, research focusing on personalized VR rehabilitation programs tailored to individual patient characteristics and preferences could optimize treatment outcomes and enhance patient satisfaction. Lastly, exploring the potential of VR technology in tele-rehabilitation and remote monitoring of KOA patients could address barriers to access and improve healthcare delivery in underserved populations.

## 5 Conclusion

This systematic review and meta-analysis suggest that exercise based on virtual reality (VR) can significantly alleviate pain, enhance muscle strength, and improve WOMAC total score and WOMAC pain in KOA patients. However, the improvement in joint stiffness and physical function is not significant. Nevertheless, given the limited number of studies, further research is necessary to expand the current analysis and provide more rigorous evidence.

## Data Availability

The original contributions presented in the study are included in the article/Supplementary Material, further inquiries can be directed to the corresponding author.

## Supplementary material

SUPPLEMENTARY FIGURE S1 Forest plots of subgroup analysis in pain.

SUPPLEMENTARY FIGURE S2 Forest plot for VR-based exercise compared with controls in WOMAC pain, stiffness, and physical function.

SUPPLEMENTARY FIGURE S3 Forest plots of subgroup analysis in WOMAC.

SUPPLEMENTARY TABLE S1 Preferred Reporting Items for Systematic Reviews and Meta-Analyses Statement (PRISMA 2020).

SUPPLEMENTARY TABLE S2 Search strategies for different databases.

SUPPLEMENTARY TABLE S3 GRADE Certainty of Evidence Evaluation.
