# Supplementary material for: Efficacy of virtual reality exercise in knee osteoarthritis rehabilitation: a systematic review and meta-analysis
Source: Front Physiol. 2024 Jun 19;15:1424815. doi: 10.3389/fphys.2024.1424815 (PMC11220424; doi:10.3389/fphys.2024.1424815)
Supplement: Supplementary file 1 [file DataSheet1.ZIP › Supplementary Table S1-4.docx]

Supplementary Material

# Supplementary Tables

**Supplementary Table S1.** Preferred Reporting Items for Systematic Reviews and Meta-Analyses Statement (PRISMA 2020).

| **Section and Topic** | **Item #** | **Checklist item** | **Location where item is reported** |
| --- | --- | --- | --- |
| **TITLE** | | |  |
| Title | 1 | Identify the report as a systematic review. | Page 1 |
| **ABSTRACT** | | |  |
| Abstract | 2 | See the PRISMA 2020 for Abstracts checklist. | Page 1, 2 |
| **INTRODUCTION** | | |  |
| Rationale | 3 | Describe the rationale for the review in the context of existing knowledge. | Page 2, 3 |
| Objectives | 4 | Provide an explicit statement of the objective(s) or question(s) the review addresses. | Page 2, 3 |
| **METHODS** | | |  |
| Eligibility criteria | 5 | Specify the inclusion and exclusion criteria for the review and how studies were grouped for the syntheses. | Page 4 |
| Information sources | 6 | Specify all databases, registers, websites, organisations, reference lists and other sources searched or consulted to identify studies. Specify the date when each source was last searched or consulted. | Page 3 |
| Search strategy | 7 | Present the full search strategies for all databases, registers and websites, including any filters and limits used. | Page 3 |
| Selection process | 8 | Specify the methods used to decide whether a study met the inclusion criteria of the review, including how many reviewers screened each record and each report retrieved, whether they worked independently, and if applicable, details of automation tools used in the process. | Page 4 |
| Data collection process | 9 | Specify the methods used to collect data from reports, including how many reviewers collected data from each report, whether they worked independently, any processes for obtaining or confirming data from study investigators, and if applicable, details of automation tools used in the process. | Page 4 |
| Data items | 10a | List and define all outcomes for which data were sought. Specify whether all results that were compatible with each outcome domain in each study were sought (e.g. for all measures, time points, analyses), and if not, the methods used to decide which results to collect. | Page 4 |
|  | 10b | List and define all other variables for which data were sought (e.g. participant and intervention characteristics, funding sources). Describe any assumptions made about any missing or unclear information. | Page 4 |
| Study risk of bias assessment | 11 | Specify the methods used to assess risk of bias in the included studies, including details of the tool(s) used, how many reviewers assessed each study and whether they worked independently, and if applicable, details of automation tools used in the process. | Page 4 |
| Effect measures | 12 | Specify for each outcome the effect measure(s) (e.g. risk ratio, mean difference) used in the synthesis or presentation of results. | Page 4, 5 |
| Synthesis methods | 13a | Describe the processes used to decide which studies were eligible for each synthesis (e.g. tabulating the study intervention characteristics and comparing against the planned groups for each synthesis (item #5)). | Page 4 |
|  | 13b | Describe any methods required to prepare the data for presentation or synthesis, such as handling of missing summary statistics, or data conversions. | Page 4, 5 |
|  | 13c | Describe any methods used to tabulate or visually display results of individual studies and syntheses. | None |
|  | 13d | Describe any methods used to synthesize results and provide a rationale for the choice(s). If meta-analysis was performed, describe the model(s), method(s) to identify the presence and extent of statistical heterogeneity, and software package(s) used. | Page 5 |
|  | 13e | Describe any methods used to explore possible causes of heterogeneity among study results (e.g. subgroup analysis, meta-regression). | Page 5 |
|  | 13f | Describe any sensitivity analyses conducted to assess robustness of the synthesized results. | Page 5 |
| Reporting bias assessment | 14 | Describe any methods used to assess risk of bias due to missing results in a synthesis (arising from reporting biases). | Page 4, 5 |
| Certainty assessment | 15 | Describe any methods used to assess certainty (or confidence) in the body of evidence for an outcome. | Page 4 |
| **RESULTS** | | |  |
| Study selection | 16a | Describe the results of the search and selection process, from the number of records identified in the search to the number of studies included in the review, ideally using a flow diagram. | Page 5, Figure 1 |
|  | 16b | Cite studies that might appear to meet the inclusion criteria, but which were excluded, and explain why they were excluded. | Page 5 |
| Study characteristics | 17 | Cite each included study and present its characteristics. | Page 5, 6 |
| Risk of bias in studies | 18 | Present assessments of risk of bias for each included study. | Page 6 |
| Results of individual studies | 19 | For all outcomes, present, for each study: (a) summary statistics for each group (where appropriate) and (b) an effect estimate and its precision (e.g. confidence/credible interval), ideally using structured tables or plots. | Page 6-8 |
| Results of syntheses | 20a | For each synthesis, briefly summarise the characteristics and risk of bias among contributing studies. | Page 5, 6 |
|  | 20b | Present results of all statistical syntheses conducted. If meta-analysis was done, present for each the summary estimate and its precision (e.g. confidence/credible interval) and measures of statistical heterogeneity. If comparing groups, describe the direction of the effect. | Page 6-8 |
|  | 20c | Present results of all investigations of possible causes of heterogeneity among study results. | Page 6,7 |
|  | 20d | Present results of all sensitivity analyses conducted to assess the robustness of the synthesized results. | Page 6,7 |
| Reporting biases | 21 | Present assessments of risk of bias due to missing results (arising from reporting biases) for each synthesis assessed. | None |
| Certainty of evidence | 22 | Present assessments of certainty (or confidence) in the body of evidence for each outcome assessed. | Page 8 |
| **DISCUSSION** | | |  |
| Discussion | 23a | Provide a general interpretation of the results in the context of other evidence. | Page 8 |
|  | 23b | Discuss any limitations of the evidence included in the review. | Page 11 |
|  | 23c | Discuss any limitations of the review processes used. | Page 11 |
|  | 23d | Discuss implications of the results for practice, policy, and future research. | Page 11, 12 |
| **OTHER INFORMATION** | | |  |
| Registration and protocol | 24a | Provide registration information for the review, including register name and registration number, or state that the review was not registered. | Page 2 |
|  | 24b | Indicate where the review protocol can be accessed, or state that a protocol was not prepared. | Page 3 |
|  | 24c | Describe and explain any amendments to information provided at registration or in the protocol. | None |
| Support | 25 | Describe sources of financial or non-financial support for the review, and the role of the funders or sponsors in the review. | Page 12 |
| Competing interests | 26 | Declare any competing interests of review authors. | Page 12 |
| Availability of data, code and other materials | 27 | Report which of the following are publicly available and where they can be found: template data collection forms; data extracted from included studies; data used for all analyses; analytic code; any other materials used in the review. | Page 12 |

**Supplementary Table S2** Search strategies for different databases.

**Search strategy for PubMed**

#1 Osteoarthritis, Knee [MeSH Terms]

#2(Knee Osteoarthritides[Title/Abstract]) OR (Knee Osteoarthritis[Title/Abstract])) OR (Osteoarthritis of Knee[Title/Abstract] OR (Osteoarthritis of the Knee[Title/Abstract])

#3 #1 OR #2

#4 Virtual Reality [MeSH Terms]

#5 Virtual Reality Exposure Therapy [MeSH Terms]

#6 (Reality, Virtual[Title/Abstract]) OR (Virtual Reality, Educational[Title/Abstract]) OR (Educational Virtual Realities[Title/Abstract]) OR (Educational Virtual Reality[Title/Abstract]) OR (Reality, Educational Virtual[Title/Abstract]) OR (Virtual Realities, Educational[Title/Abstract]) OR (Virtual Reality, Instructional[Title/Abstract]) OR (Instructional Virtual Realities[Title/Abstract]) OR (Instructional Virtual Reality[Title/Abstract]) OR (Realities, Instructional Virtual[Title/Abstract]) OR (Reality, Instructional Virtual[Title/Abstract]) OR (Virtual Realities, Instructional[Title/Abstract]) OR (Virtual Reality Immersion Therapy[Title/Abstract]) OR (Virtual Reality Therapy[Title/Abstract]) OR (Reality Therapies, Virtual[Title/Abstract]) OR (Reality Therapy, Virtual[Title/Abstract]) OR (Therapies, Virtual Reality[Title/Abstract]) OR (Therapy, Virtual Reality[Title/Abstract]) OR (Virtual Reality Therapies[Title/Abstract])

#7 #4 OR #5 OR #6

#8 (game[Title/Abstract]) OR (games[Title/Abstract]) OR (computer game[Title/Abstract]) OR (computer games[Title/Abstract]) OR (videogame[Title/Abstract]) OR (videogames[Title/Abstract]) OR (video game[Title/Abstract]) OR (video games[Title/Abstract]) OR (active game[Title/Abstract]) OR (active games[Title/Abstract]) OR (serious game[Title/Abstract]) OR (serious games[Title/Abstract]) OR (Exergam*[Title/Abstract]) OR (Interactive[Title/Abstract]) OR (immersive[Title/Abstract]) OR (Wii[Title/Abstract]) OR (Kinect[Title/Abstract]) OR (Xbox[Title/Abstract]) OR (Playstation[Title/Abstract]) OR (oculus[Title/Abstract])

#9 #7 OR #8

#10 #3 AND #9

**Search strategy for EMBASE**

#1 ‘Osteoarthritis, Knee ‘/exp

#2 'Knee Osteoarthritides':ab,ti OR 'Knee Osteoarthritis':ab,ti OR 'Osteoarthritis of Knee':ab,ti OR 'Osteoarthritis of the Knee':ab,ti

#3 #1 OR #2

#4 ‘Virtual Reality’ /exp

#5 ‘Virtual Reality Exposure Therapy’ /exp

#6 ‘Reality, Virtual’ OR ‘Virtual Reality, Educational’ OR ‘Educational Virtual Realities’ OR ‘Educational Virtual Reality’ OR ‘Reality, Educational Virtual’ OR ‘Virtual Realities, Educational’ OR ‘Virtual Reality, Instructional’ OR ‘Instructional Virtual Realities’ OR ‘Instructional Virtual Reality’ OR ‘Realities, Instructional Virtual’ OR ‘Reality, Instructional Virtual’ OR ‘Virtual Realities, Instructional’ OR ‘Virtual Reality Immersion Therapy’ OR ‘Virtual Reality Therapy’ OR ‘Reality Therapies, Virtual’ OR ‘Reality Therapy, Virtual’ OR ‘Therapies, Virtual Reality’ OR ‘Therapy, Virtual Reality’ OR ‘Virtual Reality Therapies’ OR ‘game(s)’ OR ‘computer game(s)’ OR ‘videogame(s)’ OR ‘video game(s)’ OR ‘active game(s)’ OR ‘serious game(s)’ OR ‘exergam’ OR ‘interactive’ OR ‘immersive’ OR ‘wii’ OR ‘kinect’ OR ‘xbox’ OR ‘playstation’ OR ‘oculus’

#7 #4 OR #5 OR #6

#8 #3 AND #7

**Search strategy for Web of science**

#1 TS=(Osteoarthritis,Knee OR Knee Osteoarthritides OR Knee Osteoarthritis OR Osteoarthritis of Knee OR Osteoarthritis of the Knee OR knee osteoarthritis)

#2 TS=(Virtual Reality OR Virtual Reality Exposure Therapy OR Reality, Virtual OR Virtual Reality, Educational OR Educational Virtual Realities OR Educational Virtual Reality OR Reality, Educational Virtual OR Virtual Realities, Educational OR Virtual Reality, Instructional OR Instructional Virtual Realities OR Instructional Virtual Reality OR Realities, Instructional Virtual OR Reality, Instructional Virtual OR Virtual Realities, Instructional OR Virtual Reality Immersion Therapy OR Virtual Reality Therapy OR Reality Therapies, Virtual OR Reality Therapy, Virtual OR Therapies, Virtual Reality OR Therapy, Virtual Reality OR Virtual Reality Therapies OR game* OR computer game* OR videogame* OR video game* OR active game* OR serious game* OR Exergam OR Interactive OR immersive OR Wii OR Kinect OR Xbox OR Playstation OR oculus )

#3 (#1 AND #2)

**Search strategy for The Cochrane Library**

#1 MeSH descriptor: [Osteoarthritis, Knee] explode all trees

#2 MeSH descriptor: [Virtual Reality] explode all trees

#3 MeSH descriptor: [Virtual Reality Exposure Therapy] explode all trees

#4 (Knee Osteoarthritides):ab,ti,kw OR(Knee Osteoarthritis):ab,ti,kw OR (Osteoarthritis of Knee):ab,ti,kw OR (Osteoarthritis of the Knee):ab,ti,kw

#5 (Reality, Virtual OR Virtual Reality, Educational OR Educational Virtual Realities OR Educational Virtual Reality OR Reality, Educational Virtual OR Virtual Realities, Educational OR Virtual Reality, Instructional OR Instructional Virtual Realities OR Instructional Virtual Reality OR Realities, Instructional Virtual OR Reality, Instructional Virtual OR Virtual Realities, Instructional OR Virtual Reality Immersion Therapy OR Virtual Reality Therapy OR Reality Therapies, Virtual OR Reality Therapy, Virtual Therapies, Virtual Reality OR Therapy, Virtual Reality OR Virtual Reality Therapies):ti,ab,kw (Word variations have been searched)

#6 (game(s) OR computer game(s) OR videogame(s) OR video game(s) OR active game(s) OR serious game(s) OR Exergam OR Interactive OR immersive OR Wii OR Kinect OR Xbox OR Playstation OR oculus):ti,ab,kw

#7 #1 OR #4

#8 #2 OR #3 OR #5 OR #6

#9 #7 AND #8

**Search strategy for SCOPUS**

#1 (TITLE-ABS-KEY(Reality, Virtual) OR TITLE-ABS-KEY(Virtual Reality) OR TITLE-ABS-KEY(Virtual Reality, Educational) OR TITLE-ABS-KEY(Educational Virtual Realities) OR TITLE-ABS-KEY(Educational Virtual Reality) OR TITLE-ABS-KEY(Reality, Educational Virtual) OR TITLE-ABS-KEY(Virtual Realities, Educational) OR TITLE-ABS-KEY(Instructional Virtual Realities) OR TITLE-ABS-KEY(Instructional Virtual Reality) OR TITLE-ABS-KEY(Realities, Instructional Virtual) OR TITLE-ABS-KEY(Reality, Instructional Virtual) OR TITLE-ABS-KEY(Virtual Realities, Instructional) OR TITLE-ABS-KEY(Virtual Reality Immersion Therapy) OR TITLE-ABS-KEY(Virtual Reality Therapy) OR TITLE-ABS-KEY(Reality Therapies, Virtual) OR TITLE-ABS-KEY(Reality Therapy, Virtual) OR TITLE-ABS-KEY(Therapies, Virtual Reality) OR TITLE-ABS-KEY(Therapy, Virtual Reality) OR TITLE-ABS-KEY(Virtual Reality Therapies))

#2 (TITLE-ABS-KEY(Virtual Reality Exposure Therapy) OR TITLE-ABS-KEY(game) OR TITLE-ABS-KEY(games) OR TITLE-ABS-KEY(computer game) OR TITLE-ABS-KEY(computer games) OR TITLE-ABS-KEY(videogame) OR TITLE-ABS-KEY(videogames) OR TITLE-ABS-KEY(video game) OR TITLE-ABS-KEY(video games) OR TITLE-ABS-KEY(active game) OR TITLE-ABS-KEY(active games) OR TITLE-ABS-KEY(serious game) OR TITLE-ABS-KEY(serious games) OR TITLE-ABS-KEY(Exergam*) OR TITLE-ABS-KEY(Interactive) OR TITLE-ABS-KEY(immersive) OR TITLE-ABS-KEY(Wii) OR TITLE-ABS-KEY(Kinect) OR TITLE-ABS-KEY(Xbox) OR TITLE-ABS-KEY(Playstation) OR TITLE-ABS-KEY(oculus))

#3 ( TITLE-ABS-KEY ( osteoarthritis, AND knee ) OR TITLE-ABS-KEY ( knee AND osteoarthritides ) OR TITLE-ABS-KEY ( knee AND osteoarthritis ) OR TITLE-ABS-KEY ( osteoarthritis AND of AND knee ) OR TITLE-ABS-KEY ( osteoarthritis AND of AND the AND knee ) )

#4 #1 OR #2

#5 #3 AND #4

**Search strategy for PEDro**

#1 Virtual Reality.

#2 knee osteoarthritis

#3 #1 AND #2

**Supplementary Table S3** GRADE Certainty of Evidence Evaluation.

| **Certainty assessment** | | | | | | | **№ of patients** | | **Effect** | | **Certainty** | **Importance** |
| --- | --- | --- | --- | --- | --- | --- | --- | --- | --- | --- | --- | --- |
| **№ of studies** | **Study design** | **Risk of bias** | **Inconsistency** | **Indirectness** | **Imprecision** | **Other considerations** | **virtual reality** | **placebo** | **Relative (95% CI)** | **Absolute (95% CI)** |  |  |

VAS

| 6 | randomised trials | not serious | serious | not serious | not serious | none | 165 | 168 | - | S MD **1.53 SD**  **lower**  (2.5 lower to  0.55 lower) | ⨁⨁⨁◯  Moderate | CRITICAL |
| --- | --- | --- | --- | --- | --- | --- | --- | --- | --- | --- | --- | --- |

WOMAC Total Score

| 5 | randomised trials | not serious | serious | not serious | serious | none | 125 | 128 | - | MD **14.79 SD**  **lower**  (28.26 lower  to 1.33 lower) | ⨁⨁◯◯  Low | CRITICAL |
| --- | --- | --- | --- | --- | --- | --- | --- | --- | --- | --- | --- | --- |

Muscle strength - Knee flexors

| 3 | randomised trials | serious | serious | not serious | serious | none | 64 | 59 | - | MD **1.91**  **higher**  (0.76 higher  to 3.07 higher) | ⨁◯◯◯  Very low | IMPORTANT |
| --- | --- | --- | --- | --- | --- | --- | --- | --- | --- | --- | --- | --- |

Muscle strength - Knee extensors

| 3 | randomised trials | serious | serious | not serious | serious | none | 64 | 59 | - | MD **1.54**  **higher**  (0.51 higher  to 2.56 higher) | ⨁◯◯◯  Very low | IMPORTANT |
| --- | --- | --- | --- | --- | --- | --- | --- | --- | --- | --- | --- | --- |

CI: confidence interval; MD: mean difference; SMD: standardised mean difference
