# Supplementary figures and images for: Efficacy of virtual reality exercise in knee osteoarthritis rehabilitation: a systematic review and meta-analysis
Source: Front Physiol. 2024 Jun 19;15:1424815. doi: 10.3389/fphys.2024.1424815 (PMC11220424; doi:10.3389/fphys.2024.1424815)

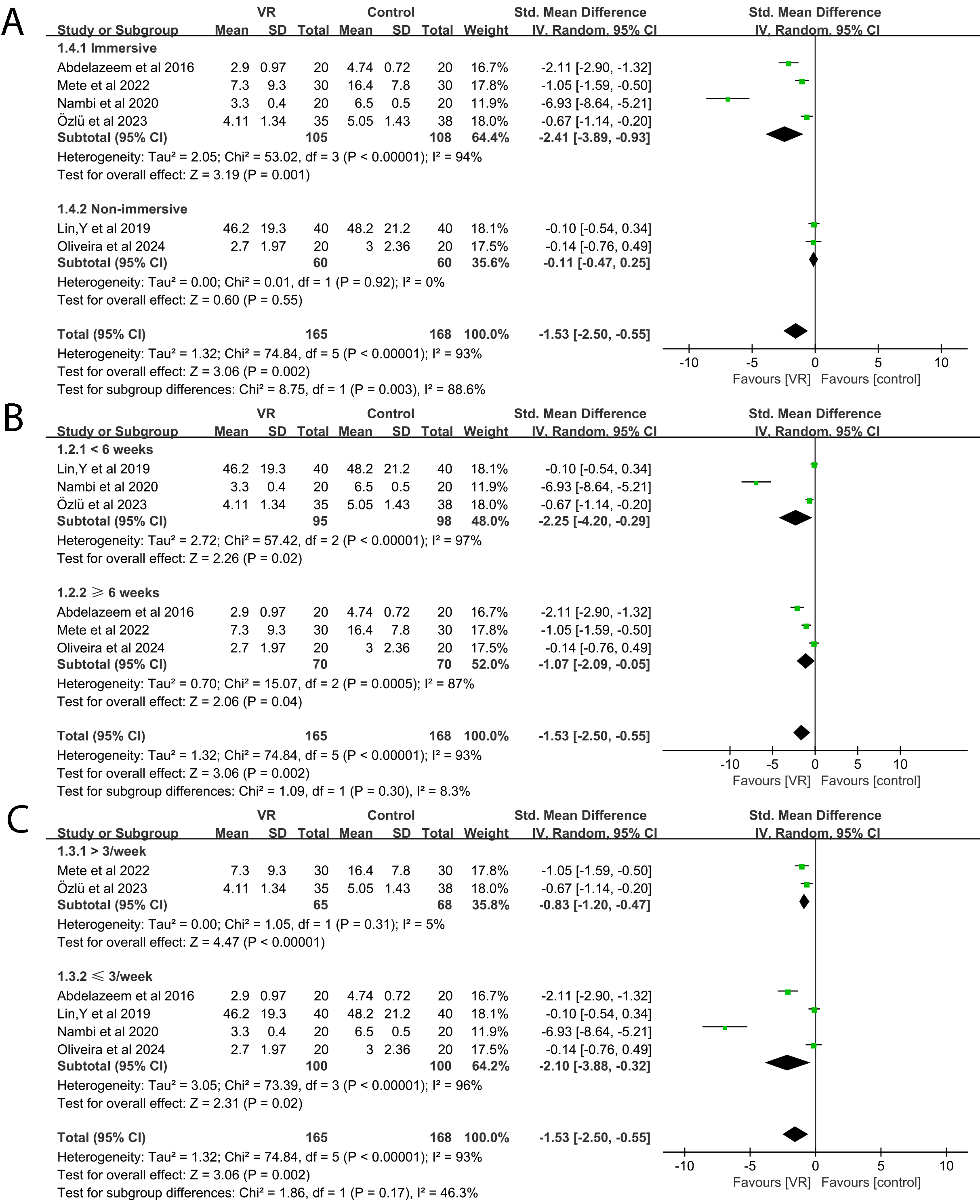

Supplement: Supplementary file 1 [file DataSheet1.ZIP › Figure S1 Forest plots of subgroup analysis in pain..tif]

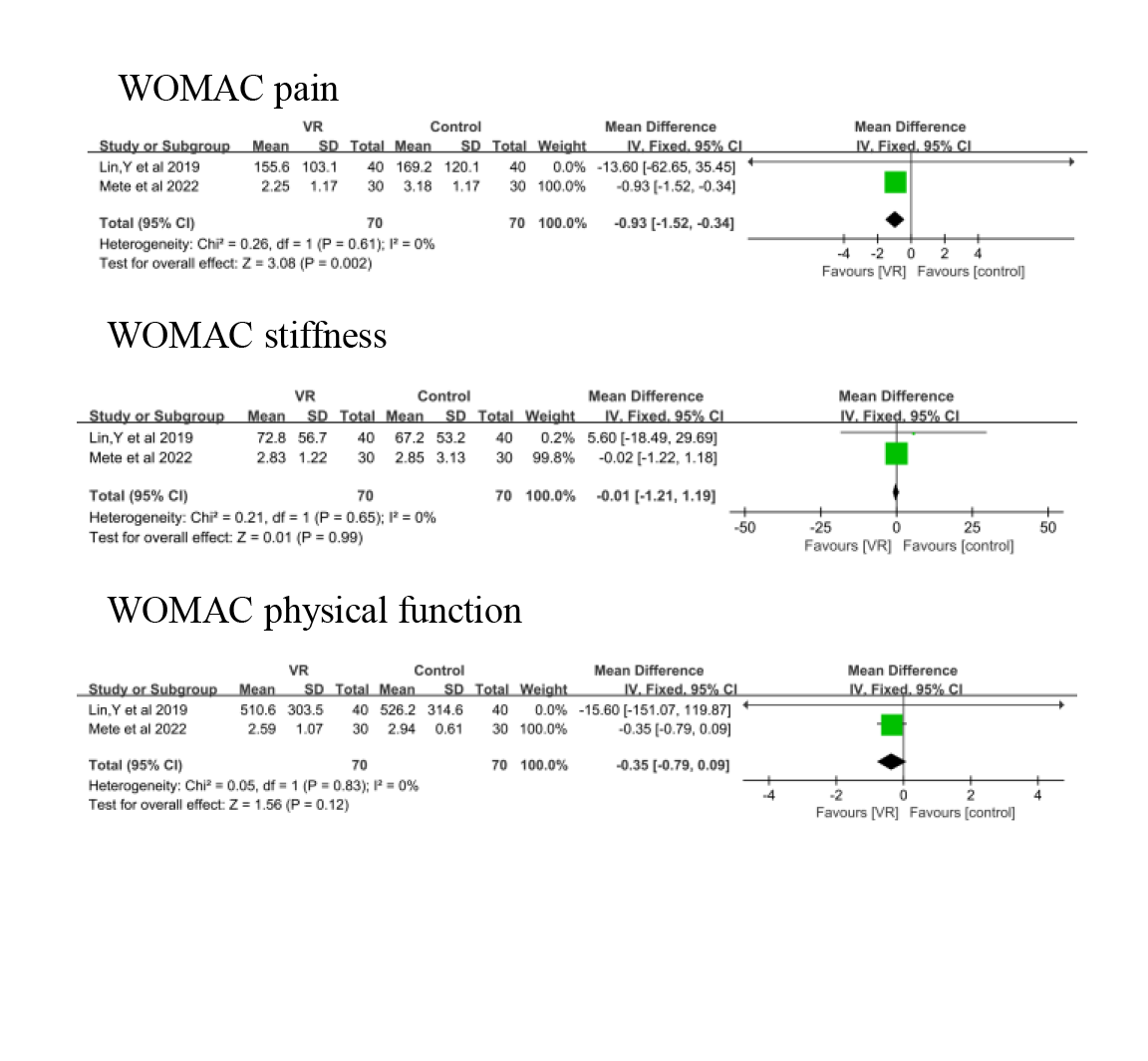

Supplement: Supplementary file 1 [file DataSheet1.ZIP › Figure S2 Forest plot for VR–based exercise compared with controls in WOMAC pain, stiffness, and physical function..tif]

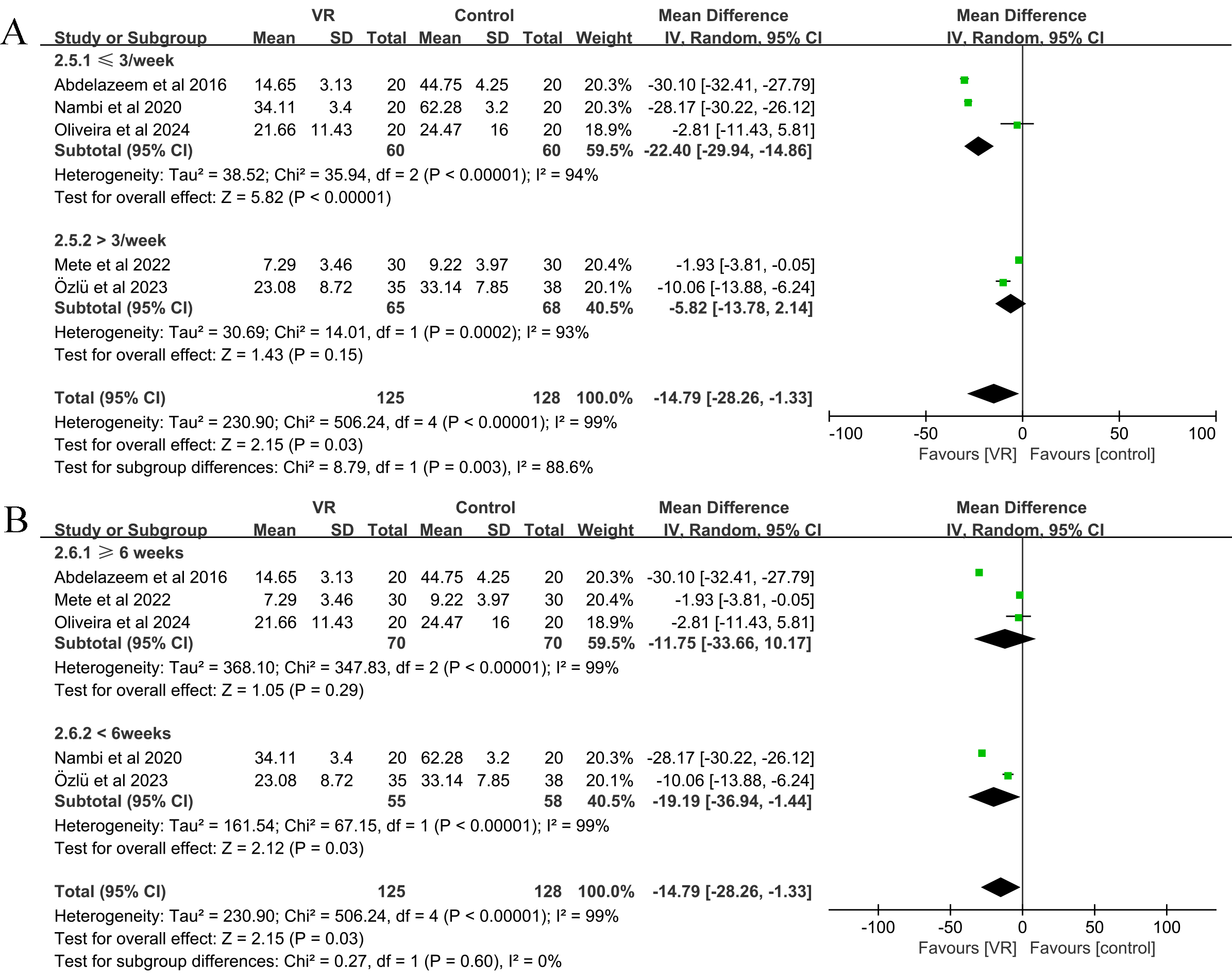

Supplement: Supplementary file 1 [file DataSheet1.ZIP › Figure S3 Forest plots of subgroup analysis in WOMAC..tif]
